# Tissue-Specific microRNA Expression Profiling to Derive Novel Biomarkers for the Diagnosis and Subtyping of Small B-Cell Lymphomas

**DOI:** 10.3390/cancers15020453

**Published:** 2023-01-10

**Authors:** Susan Swee-Shan Hue, Yu Jin, He Cheng, Muhammad Sufyan Bin Masroni, Lloyd Wei Tat Tang, Yong Howe Ho, Diana Bee-Lan Ong, Sai Mun Leong, Soo Yong Tan

**Affiliations:** 1 Department of Pathology, National University Hospital, Level 3 NUH Main Building, 21 Lower Kent Ridge Road, Singapore 119077, Singapore; 2 Department of Pathology, Yong Loo Lin School of Medicine, National University of Singapore, Level 3 NUH Main Building, 21 Lower Kent Ridge Road, Singapore 119077, Singapore; 3 MiRXES Pte Ltd., 2 Tukang Innovation Grove, JTC MedTech Hub, #08-01, Singapore 618305, Singapore; 4 Department of Pharmacy, Faculty of Science, National University of Singapore, Block S4A, Level 3, 18 Science Drive 4, Singapore 117543, Singapore; 5 Department of Pathology, Tan Tock Seng Hospital, Level 2 Podium Block, 11 Jalan Tan Tock Seng, Singapore 308433, Singapore; 6 Department of Pathology, University of Malaya, Lembah Pantai, Kuala Lumpur 50603, Malaysia; 7 Advanced Molecular Pathology Laboratory, Institute of Molecular and Cell Biology, Agency for Science, Technology and Research (A*STAR), 61 Biopolis Drive, Proteos, Singapore 138673, Singapore

**Keywords:** small B cell lymphoma, microRNA, biomarker, diagnosis, molecular subtyping

## Abstract

**Simple Summary:**

It is highly challenging for pathologists to distinguish small B-cell lymphomas from reactive lymphoid tissue and to accurately diagnose common histological subtypes of such lymphomas. This is due to overlapping morphological features and limitations of current ancillary testing, which itself involves many further tests. Hence, there is a pressing need for better biomarkers for accurate diagnosis and subtyping of small B-cell lymphomas as better diagnosis can lead to better treatments and clinical outcomes for patients. In this study, we identified and validated two sets of microRNA biomarkers that can distinguish small B-cell lymphomas from reactive lymphoid tissue and distinguish between four subtypes of such lymphomas, respectively. This study suggests that miRNA expression profiling may serve as a promising tool to aid in the diagnosis of small B-cell lymphomas.

**Abstract:**

Accurate diagnosis of the most common histological subtypes of small B-cell lymphomas is challenging due to overlapping morphological features and limitations of ancillary testing, which involves a large number of immunostains and molecular investigations. In addition, a common diagnostic challenge is to distinguish reactive lymphoid hyperplasia that do not require additional stains from such lymphomas that need ancillary investigations. We investigated if tissue-specific microRNA (miRNA) expression may provide potential biomarkers to improve the pathology diagnostic workflow. This study seeks to distinguish reactive lymphoid proliferation (RL) from small B-cell lymphomas, and to further distinguish the four main subtypes of small B-cell lymphomas. Two datasets were included: a discovery cohort (*n* = 100) to screen for differentially expressed miRNAs and a validation cohort (*n* = 282) to develop classification models. The models were evaluated for accuracy in subtype prediction. MiRNA gene set enrichment was also performed to identify differentially regulated pathways. 306 miRNAs were detected and quantified, resulting in 90-miRNA classification models from which smaller panels of miRNAs biomarkers with good accuracy were derived. Bioinformatic analysis revealed the upregulation of known and other potentially relevant signaling pathways in such lymphomas. In conclusion, this study suggests that miRNA expression profiling may serve as a promising tool to aid the diagnosis of common lymphoid lesions.

## 1. Introduction

Lymphoma diagnosis is well recognized as one of the most difficult areas of diagnostic pathology. The pathological diagnosis of lymphoma hinges largely upon recognition of morphological abnormalities on a well-represented tissue section. However, to many pathologists who have limited experience in examining lymphoid tissues, identification of normal and pathological changes in different lymphoid compartments and recognition of neoplastic lymphoid entities may be highly challenging. Lymphoid tissue often appears to be a morass of small and large lymphoid cells that defies recognition of cell types and functional compartmentalization. Compounding this is the complexity of lymphoma classification, often necessitating the incorporation of additional testing with a plethora of immunostains and molecular genetic investigations for definitive diagnosis, making lymphoma diagnosis one of the most complicated tasks encountered by pathologists worldwide.

Therefore, it is not surprising that errors in lymphoid tissue diagnosis are prevalent [1]. Misdiagnoses of reactive lymphoid proliferation from neoplastic ones (and vice versa) and misclassification of neoplastic lymphoid entities can have serious consequences related to inappropriate treatments being administered to the patients. In this regard, differentiating reactive lymphoid proliferations from their mature, small-sized, or low-grade B-cell neoplastic counterparts (henceforth collectively termed small B-cell lymphomas) is particularly problematic. Small B-cell lymphomas comprise a heterogeneous admixture of small and occasionally larger lymphoid cells with only mild cytologic atypia, and some cases may even retain the tissue architecture to some degree, therefore resembling reactive lymphoid proliferations to the lesser trained eye. There is also significant morphological overlap between the different subtypes of these small B-cell lymphomas, making immunohistochemistry (IHC) and/or molecular genetic testing an integral component in the proper workup of these neoplasms [2]. Given the increasing gravitation towards small needle core biopsy that renders limited tissue samples, the lack of access to an adequate range of IHC in smaller hospitals as well as the lack of familiarity with ancillary molecular testing, many pathologists frequently encounter tremendous difficulties in making a confident diagnosis of such lymphoid proliferations. All the above emphasize a need for additional ancillary tools that employ fewer tissue sections, provide objective data, and are preferably low-cost for the diagnostic workup of small B-cell lymphomas.

In this study, we investigated the potential utility of microRNA (miRNA) expression signatures as an adjunctive ancillary test in the diagnosis and classification of small B-cell lymphomas. MiRNAs, a family of short, evolutionarily conserved, non-coding RNA of approximately 18–25 nucleotides [3,4], have shown great promise as novel diagnostic biomarkers. Apart from their involvement in normal physiological processes as a master regulator of post-transcriptional gene expression [5,6], they have also been shown to play essential roles in the development of cancer phenotypes [7,8]. Given their high stability in clinical tissue samples, tumor tissue miRNA expression profiles are emerging as an attractive tool to aid tumor classification in line with their cellular lineage, differentiation state, and molecular alterations. To achieve this end, we used a novel, high-throughput, quantitative real-time PCR (qPCR) platform [9,10] to profile miRNA expression of the four most common small B-cell lymphoma entities, namely small lymphocytic lymphoma/chronic lymphocytic leukemia (SLL), low-grade follicular lymphoma (FL), mantle cell lymphoma (MCL) and marginal zone lymphoma (MZL), and compared them with that of reactive lymphoid proliferations (RL) from various nodal and extranodal tissue sites. We aimed to evaluate whether the levels of miRNA from FFPE patient samples (i) can be used to differentiate cases of RL from lymphoma and (ii) can effectively distinguish between cases of SLL, FL, MCL and MZL.

## 2. Materials and Methods

### 2.1. Experimental Design

Formalin-fixed paraffin-embedded (FFPE) tissue samples from RL and the 4 histological subtypes of small B-cell lymphomas were included: SLL, FL, MCL, and MZL. The diagnosis was based on the criteria established in the World Health Organization (WHO) classification [11]. Samples recruited comprise both excisional and core tissue biopsies from nodal and extranodal sites (Table 1). All cases were reviewed by pathologists with experience in haematolymphoid pathology to verify the diagnosis. Institutional Review Board approval was obtained for all samples in accordance with the NHG’s Institutional Review Board (IRB) Guidelines. 

A total of 382 subjects were included in this study. A discovery set of 100 FFPE tissue samples was obtained from the Department of Pathology, National University Hospital (NUH), Singapore: SLL (*n* = 23), FL (*n* = 21), MCL (*n* = 20), MZL (*n* = 19), and RL (*n* = 17). Whole tissue sections of the recruited samples were used, and the percentage of tumor cells was estimated to be 50% or more for each sample. A validation set of 282 FFPE tissue samples comprising SLL (*n* = 20), FL (*n* = 74), MCL (*n* = 22), MZL (*n* = 74), and RL (*n* = 92) was further collected from three different institutions: NUH, Tan Tock Seng Hospital (TTSH), Singapore and University Malaya Medical Centre (UMMC). Samples were classified and analyzed using miRNA expression profiling and the results were compared to the reference diagnosis.

### 2.2. RNA Isolation, Reverse-Transcription, cDNA Amplification, and Real-Time qPCR

Total RNAs were isolated from FFPE tissues using the miRNeasy FFPE miRNA isolation kit (Qiagen, Germany) according to the manufacturer’s protocol. Three synthetic short RNA species (spike-ins) with sequences distinct from endogenous human miRNAs were added into the lysis buffer as controls to monitor and normalize for workflow variations. The miRNA was eluted using 50 µL nuclease-free water. Total RNA quantity and quality were measured by NanoDrop 2000 (Thermo-Fisher Scientific, Waltham, MA, USA). For each sample, 900 ng total RNA was used for subsequent reverse-transcription and PCR reactions. 

MiRNA profiling was performed using a multiplexed RT-qPCR platform following an established protocol [10]. Isolated miRNAs underwent reverse transcription using the in-house reverse transcription system and modified stem-loop RT primer pools (MiRXES, Singapore) on a Veriti^TM^ Thermal Cycler (Applied Biosystem, Waltham, MA, USA) according to the manufacturer’s instructions. For each RT reaction, a standard panel comprising a series of six 10-fold dilutions of synthetic miRNA and two no-template controls (NTCs) were included on the same plate. cDNA was then pre-amplified using a 14-cycle PCR reaction with Augmentation Primer Pools (MiRXES, Singapore) on the Veriti^TM^ Thermal Cycler. Single qPCR was performed on the amplified cDNA samples using a miRNA-specific qPCR assay and ID3EAL miRNA qPCR Master Mix according to the manufacturer’s instruction (MiRXES, Singapore). The qPCR reaction for each sample was performed with technical duplicates on the QuantStudio 5 Real-Time PCR System (Applied Biosystem, Waltham, MA, USA). Raw threshold cycle (Ct) values were calculated using the QuantStudio^TM^ Design and analysis software with an automatic baseline setting and a threshold of 0.4. 

The synthetic spike-ins added at various stages were used to correct for variations in RNA isolation and RT-qPCR efficiency. The Ct values generated from the panel of 6 serially diluted synthetic miRNAs were then used to generate a standard curve. The absolute expression of each miRNA (number of copies present) was calculated by interpolation of the standard curve. MiRNA with a Ct value higher than NTCs (no template controls) was deemed undetectable and removed from the analysis. 

### 2.3. Data Processing and Statistical Analysis

miRNA expression levels were calculated as log_2_ copy numbers in each sample. In the discovery cohort, miRNAs that were not detected in >10% of samples were removed, resulting in 306 miRNAs in 100 samples. Biological normalization was performed by the global normalization approach demonstrated by Mestagh et al. [12], followed by Z-score standardization of each miRNA’s expression. For each subtype, Student’s *t*-test was used to compare the miRNA expression between the subtype of interest and other subtypes (one-vs.-rest). The top 3 up- and 3 down-regulated miRNAs were selected based on the most significant *p*-value, resulting in a total of 30 subtype biomarkers for five subtypes. For each pair of the five subtypes, the same Student’s *t*-test was performed and the top 3 up- and 3 down-regulated miRNAs between the two subtypes (one-vs.-one) were identified, adding up to 60 miRNA markers that differentiated the two subtypes. Finally, 10 miRNAs that displayed the lowest variance across samples were selected as the housekeeping miRNAs. A 100-miRNA customized panel including 30 one-vs.-rest biomarkers, 60 one-vs.-one markers, and 10 housekeeping miRNAs was constructed and used for the FFPE samples in the validation cohort. 

RNA from additional 282 FFPE tissues were profiled using the 100-miRNA customized panel and all expression levels were obtained as log_2_ copies/sample. The mean expression level of the 10 housekeeping miRNA was used to normalize both the discovery and validation cohort to ensure comparability. As the FFPE samples in the validation cohort were collected from three different sites (NUH, TTSH, and UMMC), the batch correction was performed using the ComBat approach [13], setting the collection site as the batch variable and including the tissue site (nodal/extra-nodal) and histology subtypes (RL, FL, MZL, MCL, SLL) as covariates. After normalization and batch correction, Student’s *t*-test was used to perform a one-vs.-rest and pairwise comparison for each subtype in the validation cohort. For all the 90 previously identified markers, the expression differences between the two groups of interest in the validation cohort were compared with the findings in the discovery cohort. miRNA markers that showed the same trend of expression changes were considered successfully validated.

### 2.4. Subtype Classification and Machine Learning

Housekeeping gene normalization was applied to the raw expression levels in both discovery and validation cohorts, followed by ComBat batch correction [13] for different collection sites in the validation dataset as described above. Batch effects between the discovery and validation cohorts were also corrected. The expression and tissue site (nodal/extra-nodal) data from the two cohorts were combined to develop a multi-marker panel for accurate classification of different subtypes. Categorical data such as tissue site was converted to numerical integers (0 and 1) for ease of analysis.

Using the combined dataset, we first developed a classification model to differentiate reactive control (RL) and lymphoma (FL, MZL, MCL, and SLL) samples. The combined dataset was subjected to 100 iterations of 4-fold cross-validation in which 3 folds were used for training and the last fold was used for testing. Support vector machine (SVM) with radial kernel was used for model training in the training datasets, and 3-fold cross-validation was performed to tune the cost parameter from 1 to 10. We applied the best-tuned model to the testing dataset, and a confusion matrix was created based on the predicted types (reactive or cancer) vs. actual samples’ types. The model’s performance was assessed by the prediction accuracy on the testing set [14]. 

Using the miRNA expression and tissue site data from the lymphoma tissue samples, we further trained classification models to differentiate the four lymphoma subtypes FL, MCL, MZL, and SLL. Similarly, 100 iterations of 4-fold cross-validation were applied to the combined dataset. One-vs.-one classification strategy was used for the multi-class classification, by employing SVM model with radial kernel to the training set for model building, and another 3-fold cross-validation was performed for the determination of the optimal cost parameter from 1 to 10 based on the highest cross-validated accuracy [15]. The best-tuned model was applied to the testing data and the model performance was evaluated by the accuracy of subtype prediction. Similarly, a confusion matrix was created based on the predicted and the actual subtypes in the testing set.

### 2.5. Pathway Analysis of Differentially Expressed miRNAs

Pathway analysis was performed with all samples pooled together through miRSEA method. Briefly, miRNA and mRNA linkages were curated with miRTarBase Release 7.0 based solely on strong experimental evidence support. Pathway database was curated from Broad Institute C2 pathways sets including Kegg, Reactome, Pathway Interaction Database and Biocarta. miRNA and pathway were correlated together by identifying the specific strength of the miRNA targeting the pathway. The *p*-value for hypergeometric distribution was used to calculate the enrichment of miRNA targets in any given pathway in the universe of targets. MiRNA fold change together with the *p*-value for pathway targeting was used to calculate the regulation of pathway with weighted Kolmogorov–Smirnov-like statistics. Pathways targeted by less than 10 miRNAs or more than 500 miRNAs were ignored. The *p*-value of any pathway regulation was calculated by randomly permutating miRNAs 10,000 times. False discovery rate correction was carried out by using the null distribution of all pathways and the enrichment of unpermutated dataset. 

## 3. Results

### 3.1. Diagnostic Challenges of Small B-Cell Lymphomas

**Figure 1:**
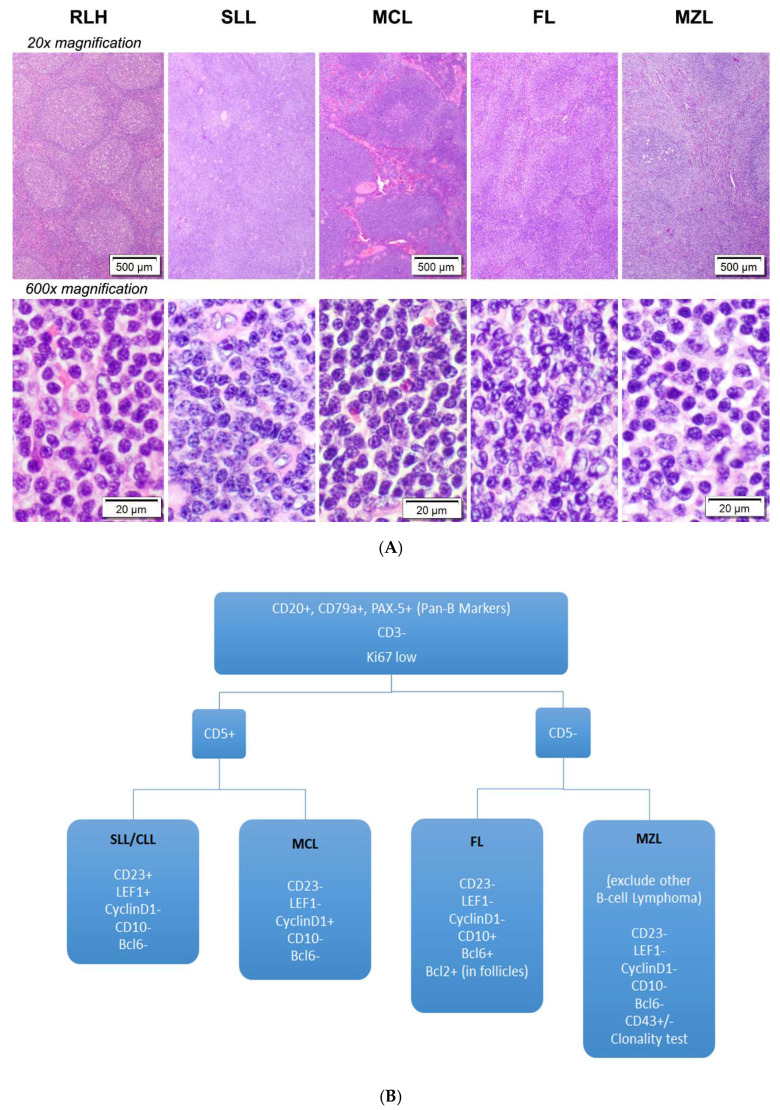
Challenges in diagnosing small B-cell lymphoma. (**A**) The morphologic overlap between reactive lymphoid hyperplasia and the four histological subtypes of small B-cell lymphomas (SLL, MCL, FL and MZL) presents considerable challenge to traditional morphological diagnosis and subtyping. Classification of lymphoma from reactive lymphoid hyperplasia often relies heavily on ancillary techniques such as immunohistochemistry. (**B**) A wide panel of immunohistochemical stains is often used by pathologists to diagnose and subtype small B-cell lymphomas. Abbreviations: RLH, reactive lymphoid hyperplasia; SLL, small lymphocytic lymphoma/chronic lymphocytic leukemia; CLL, chronic lymphocytic leukemia; MCL, mantle cell lymphoma; FL, low-grade follicular lymphoma; MZL, marginal zone lymphoma.

Current gold standard for lymphoma diagnosis is based on histopathological evaluation where tissue morphology is the foundation. However, morphological similarities between common subtypes of small B-cell lymphomas, which predominantly consist of small lymphoid cells with condensed chromatin (Figure 1A), mandate the integration of immunohistochemistry (IHC) to reach a diagnosis. However, the limited specificity and sensitivity of individual IHC marker invariably necessitate a large panel of immunostains to be used (Figure 1B), which in turn increases the diagnostic cost and amount of tissue sections required.

### 3.2. Consistency of miRNAs Expression across FFPE Samples in the Discovery and Validation Cohorts

**Figure 2:**
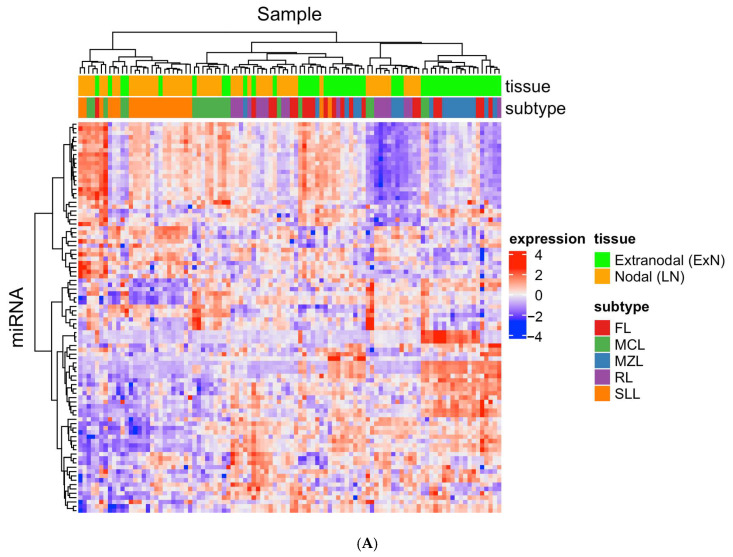

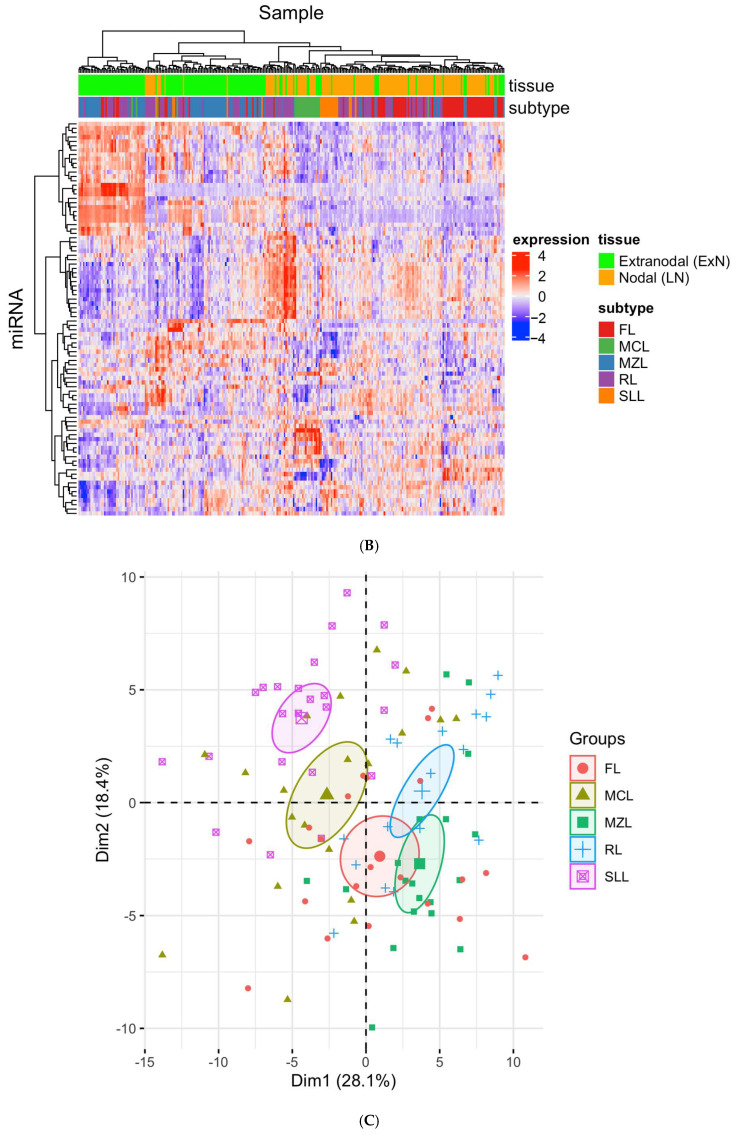

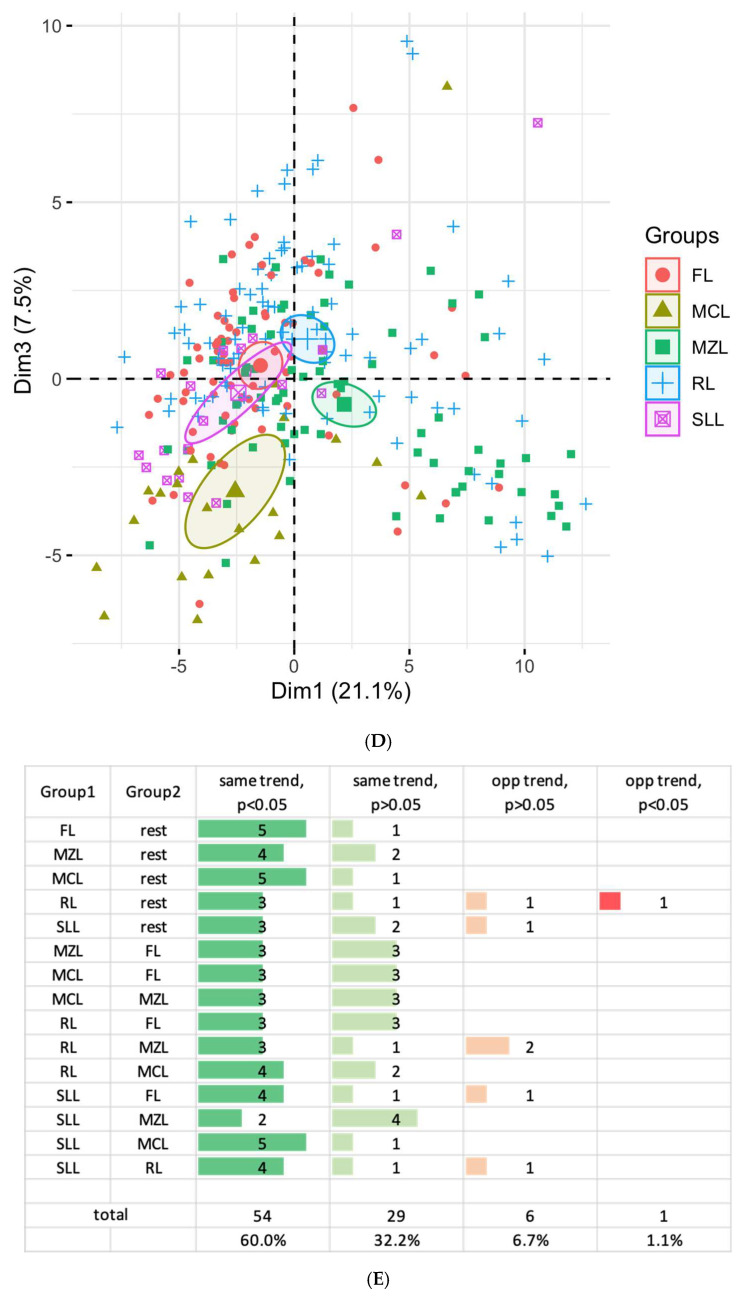

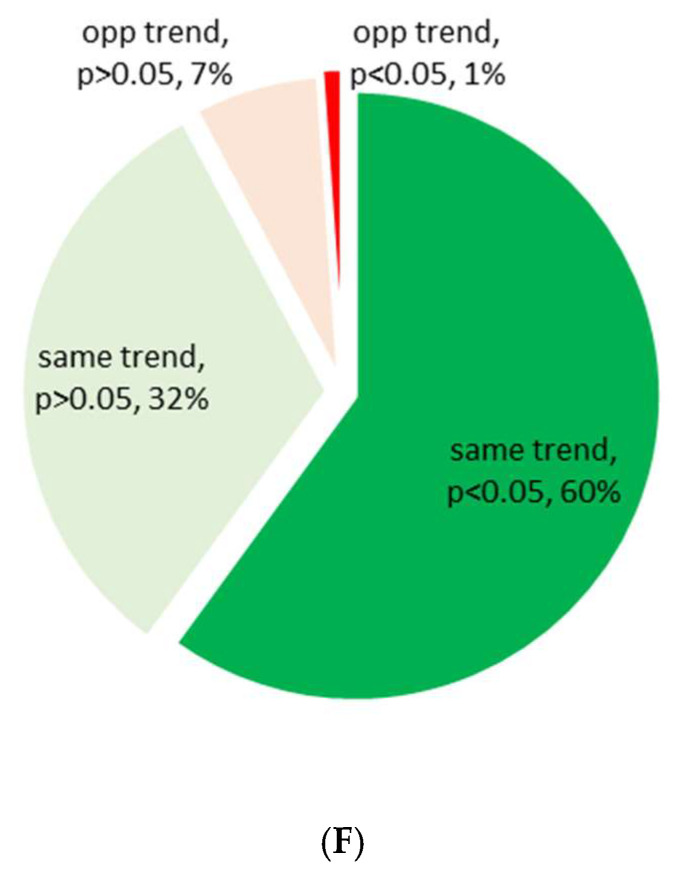
Distinct lymphoma subtypes can be distinguished by miRNA expression profiles in both discovery and validation cohorts. Heatmap showing hierarchical clustering of FFPE samples of 5 different subtypes in the discovery cohort (**A**) and validation cohort (**B**) based on the expression of 90 candidate miRNAs. Principal component analysis of miRNA expression profiles of FFPE samples in the discovery (**C**) and validation (**D**) cohort. (**E**) Comparison of the expression changes of the 90 candidate miRNAs between the discovery and validation cohorts. (**F**) Numbers of miRNAs showing consistent or inconsistent changes between discovery and validation cohorts. Abbreviations: SLL, small lymphocytic lymphoma/chronic lymphocytic leukemia; CLL, chronic lymphocytic leukemia; FL, low-grade follicular lymphoma; MCL, mantle cell lymphoma; MZL, marginal zone lymphoma; RL, reactive lymphoid proliferations.

To assess miRNAs for their potential utility as diagnostic biomarkers for diagnosis and subtyping of small B-cell lymphomas using routine FFPE samples, we first profiled the expression of 306 miRNAs in a discovery cohort of 100 samples. Out of the 306 miRNAs quantified, a 100-miRNA profiling panel comprising 10 housekeeping miRNAs and 90 candidate miRNAs was assembled for validation in the second cohort of 282 samples. Hierarchical clustering performed using the expression of the 90 candidate miRNAs showed distinct clusters representing the four subtypes of small B-cell lymphomas and RL in both the discovery and validation cohorts (Figure 2A,B). Principal component analysis (PCA) captured 46% (PC1/2) and 28% (PC1/3) of the total variance in miRNA expression profiles in the discovery and validation cohorts, respectively (Figure 2C). In the discovery cohort, the SLL and MCL subtypes can be well discriminated from other subtypes (Figure 2C), whereas in the validation cohort, MCL and MZL are the more distinct subtypes. Most of the candidate miRNAs showed robustness and consistency across the discovery and validation cohorts, with 83 out of 90 candidate miRNAs showing the same trend in pairwise comparisons (Figure 2E,F).

### 3.3. miRNAs Signature can Differentiate Lymphoma from Reactive Lymphoid Proliferation

**Figure 3:**
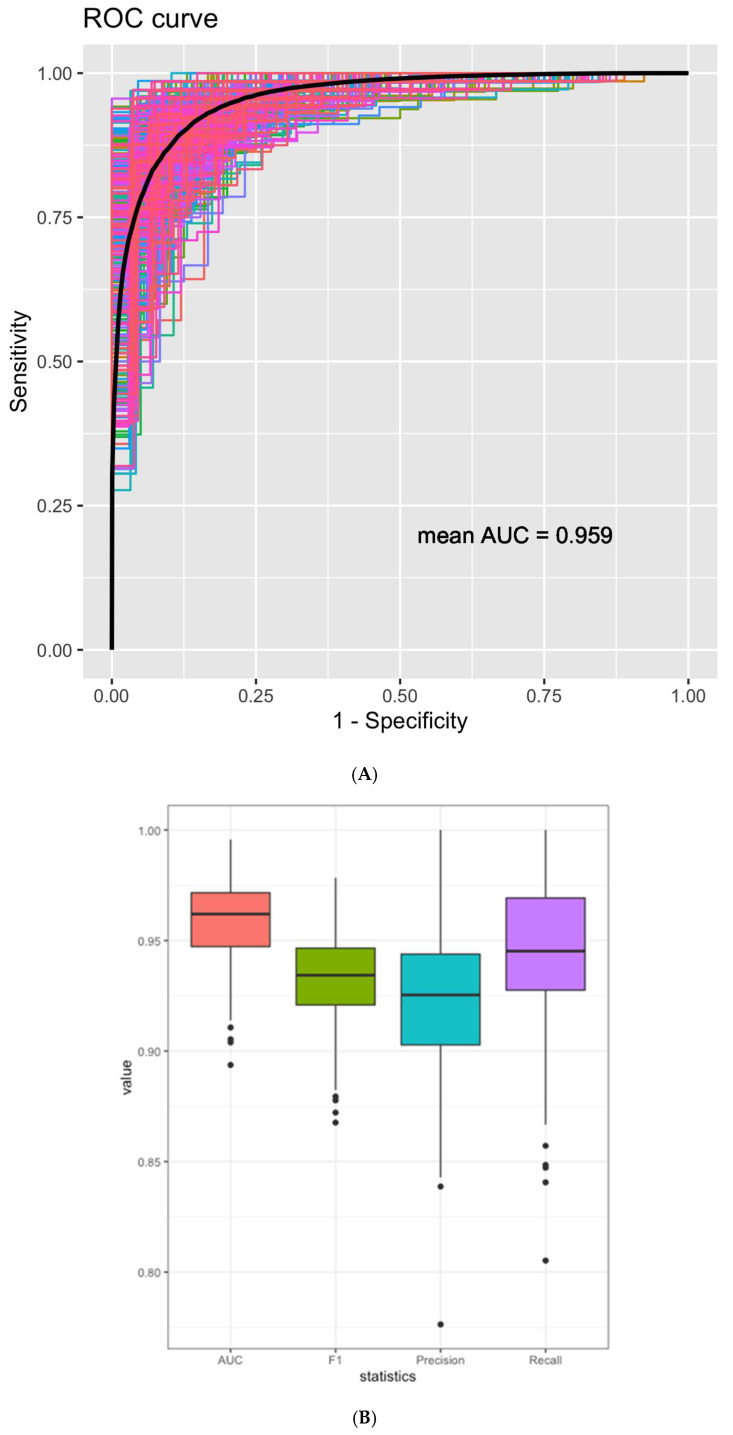

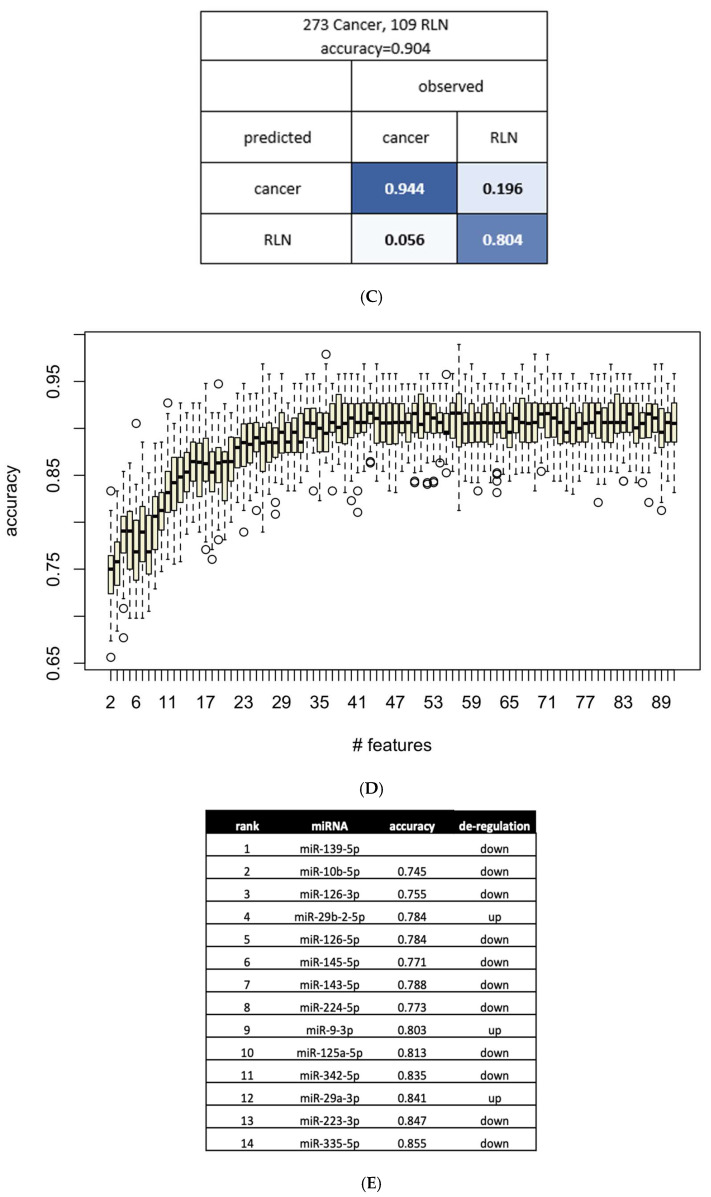
MiRNA expression as a classifier to distinguish lymphoma from reactive lymphoid proliferation (RL). (**A**) ROC curves from the test dataset of 100 times of four-fold cross-validation using the 90-miRNA classification model to differentiate lymphoma samples and RL. The black line indicates the average ROC curve. (**B**) Performance metrics of the classification model to distinguish lymphoma from RL. (**C**) Confusion matrix of the classification model from 100 times of four-fold cross-validation. (**D**) Classification accuracy for distinguishing lymphoma and RL with respect to the increasing number of miRNA features included in the classification model. (**E**) Top 14 miRNAs that achieve 85.5% classification accuracy in distinguishing between lymphoma and RL. Abbreviations: ROC, receiver operating characteristic; AUC, area under the curve.

To differentiate lymphoma from reactive lymphoid proliferation, a 90-miRNA and tissue information classification model was established by training and testing on a combined cohort of samples, resulting in a mean area under the ROC curve (AUC) of 0.959 (95% CI: 0.922 to 0.988) (Figure 3A). Other performance metrics include a mean recall of 0.944, precision of 0.923 and F1 score of 0.933 (Figure 3B). The resulting classification model has a sensitivity of 94% for lymphoma and 80.4% for reactive lymphoid proliferation, and overall accuracy of 90.4% (Figure 3C). A smaller panel comprising the top 14 miRNA features can achieve an accuracy of 85.5%, while the addition of more miRNAs did not substantially improve the accuracy (Figure 3D,E). Among the top 14 miRNA biomarkers, three were upregulated and the rest were downregulated.

### 3.4. miRNAs Signature can Subtype Small B-Cell Lymphomas

**Figure 4:**
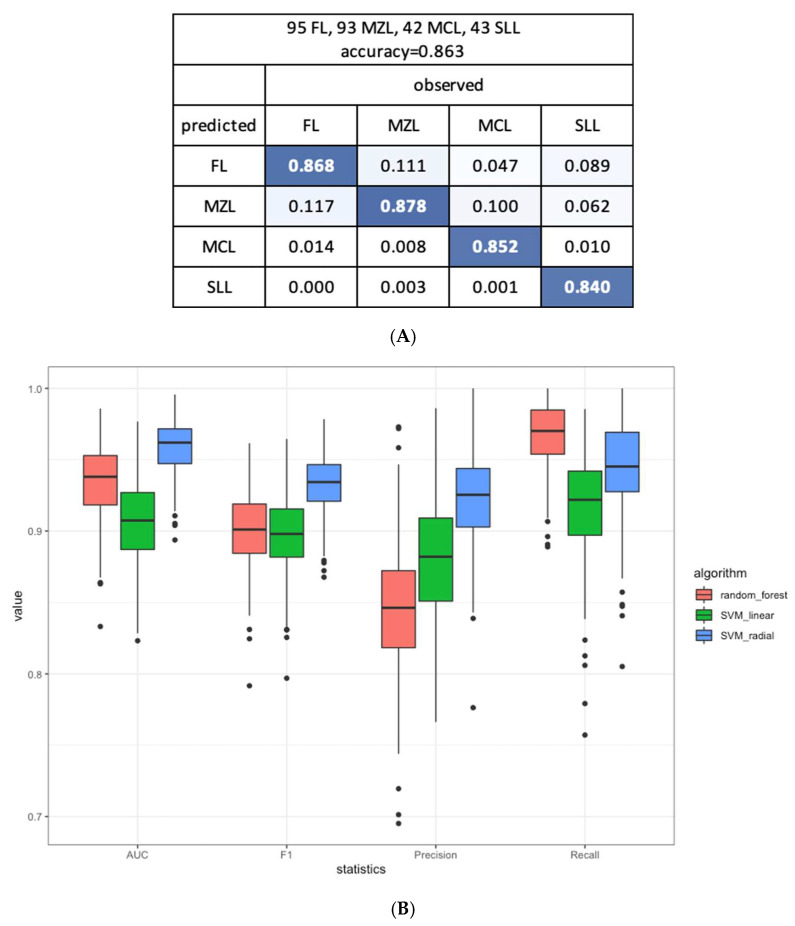

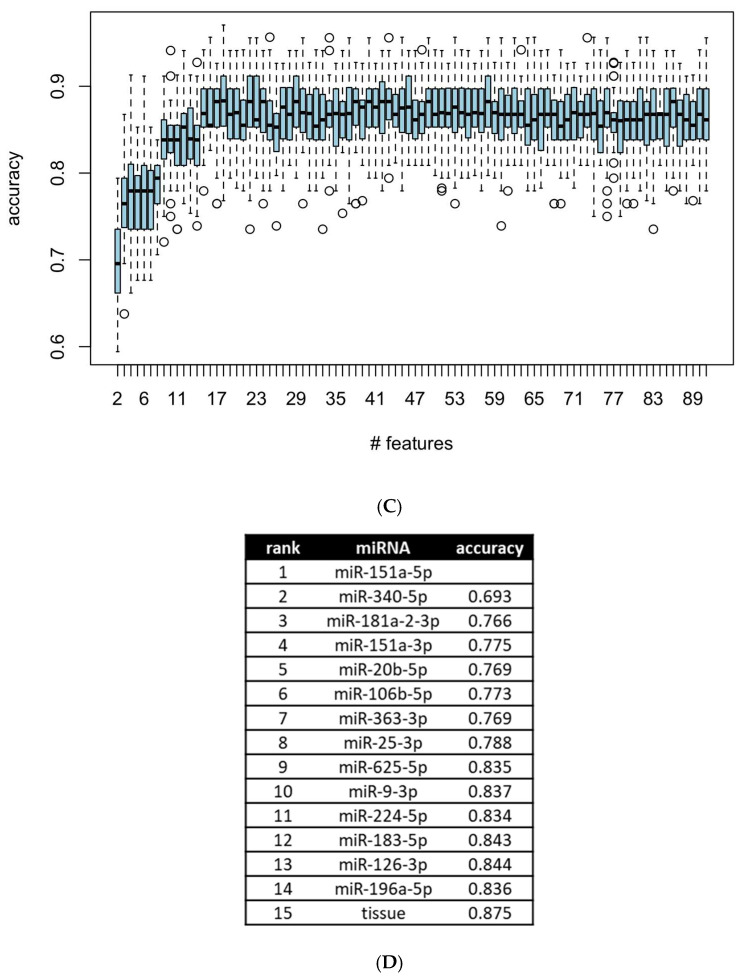
MiRNA expression as a classifier to distinguish 4 subtypes of small B-cell lymphoma. (**A**) Confusion matrix based on the average classification performance of four lymphoma subtypes in the test datasets of 100 iterations of four-fold cross-validation. (**B**) Performance comparison between three algorithms (random forest, SVM with linear kernel and SVM with radial kernel) in training the classification model. (**C**) Classification accuracy for distinguishing between the four subtypes with respect to the increasing number of miRNA features included in the classification model. (**D**) Top 14 miRNAs and tissue information that achieves 87.5% classification accuracy for four lymphoma subtypes. Abbreviations: SLL, small lymphocytic lymphoma/chronic lymphocytic leukemia; CLL, chronic lymphocytic leukemia; FL, low-grade follicular lymphoma; MCL, mantle cell lymphoma; MZL, marginal zone lymphoma; RL, reactive lymphoid proliferations.

In addition to distinguishing neoplastic from reactive lymphoid proliferation, we explored if miRNA expression profile could also be used for subtyping lymphomas. To further differentiate the four subtypes of small B-cell lymphoma, a 90-miRNA and tissue information classification model was built by training and testing using samples from both cohorts, resulting in a sensitivity of 86.8% for FL,87.8% for MZL, 85.2% for MCL and 84% for SLL and overall accuracy of 86.3% (Figure 4A). We selected the SVM with the radial kernel algorithm to build the classification model as it showed the best performance as compared to the random forest and SVM with linear kernel algorithms (Figure 4B). A smaller panel comprising the top 15 features (14 miRNAs and tissue information) can achieve an accuracy of 87.5%, while the addition of more features did not substantially improve the accuracy (Figure 4C,D). 

### 3.5. miRNA Expression could Infer Meaningful Biological Differences between Reactive and Neoplastic Lymphoid Proliferation

**Figure 5:**
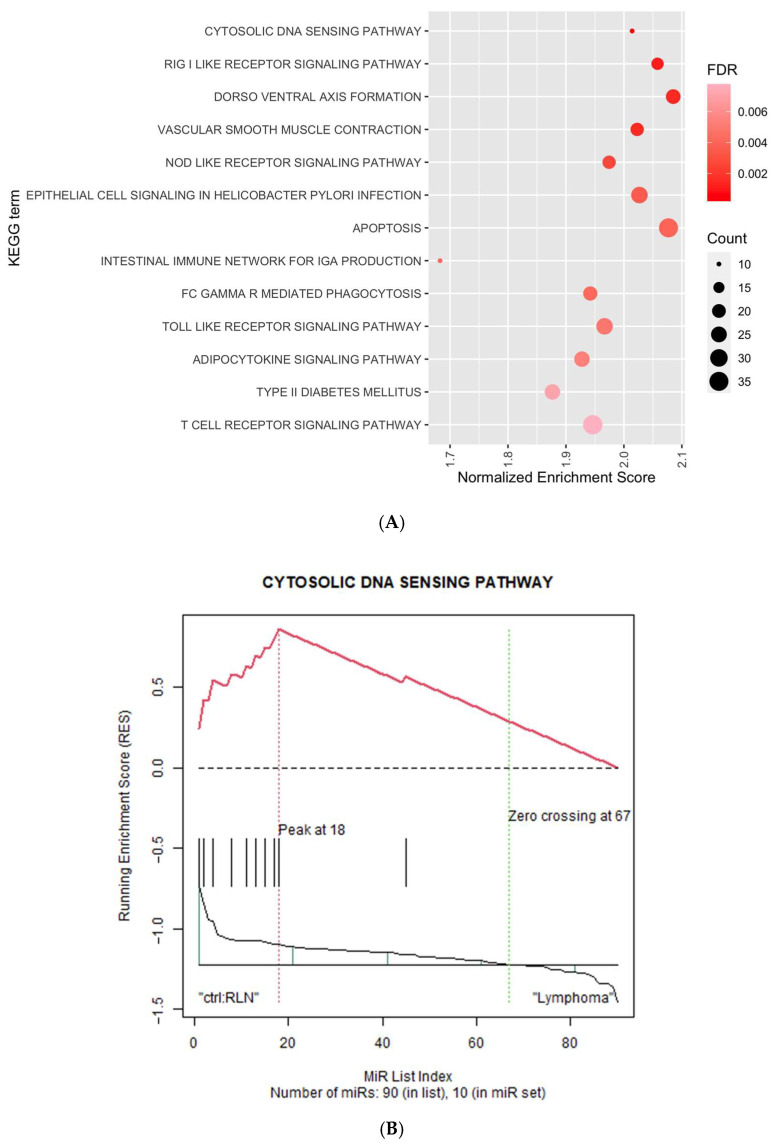

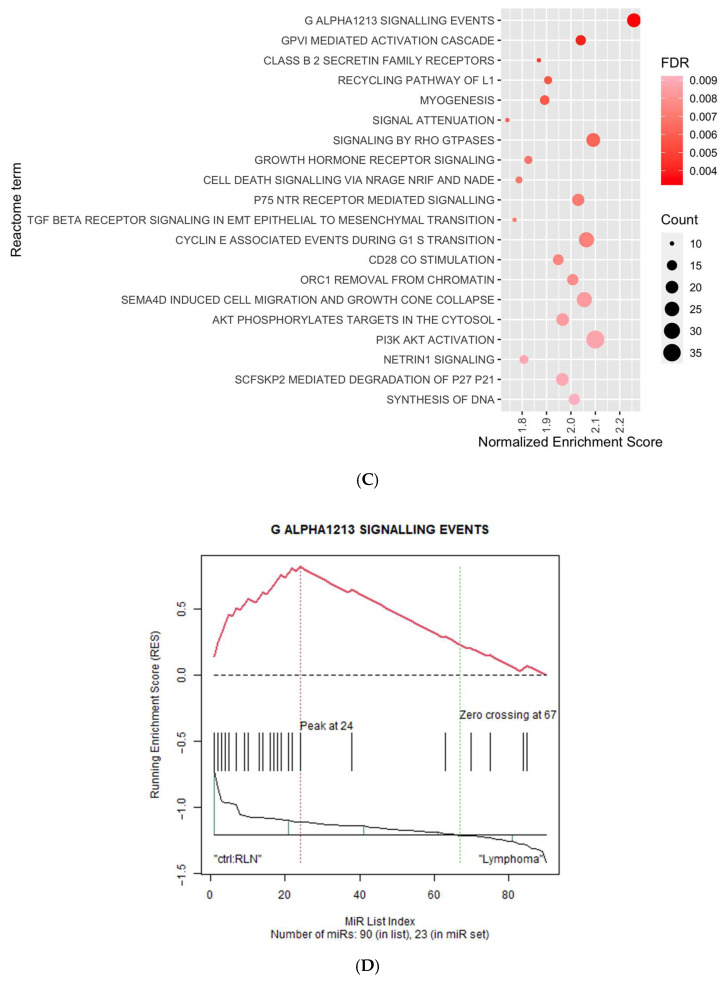
Differentially regulated pathways between lymphomas and reactive lymphoid proliferation based on deregulated miRNAs using MiRSEA analysis. (**A**) KEGG pathways significantly up-regulated in lymphoma compared to reactive lymphoid proliferation via miRNA regulation. (**B**) Cytosolic DNA sensing pathway as the most significantly enriched KEGG pathway for miRNAs de-regulated between lymphoma and RL groups. (**C**) Reactome pathways are significantly up-regulated in lymphoma compared to RL via miRNA deregulation. (**D**) Gɑ_12/13_ signaling events as the most significantly enriched reactome pathway for miRNAs de-regulated between lymphoma and RL groups. Abbreviations: KEGG, Kyoto encyclopedia of genes and genomes; FDR, false discovery rate.

To gain insights into B-cell lymphomagenesis and uncover significant signalling pathways, pathway analysis via miRNA gene set enrichment (miRSEA) was performed to identify differentially regulated pathways between lymphoma and reactive lymphoid tissues. Using a cutoff q-value of 0.01, 13 KEGG pathways (Figure 5A) and 20 Reactome pathways (Figure 5C) were found to be up-regulated in lymphoma as compared to RL. The most significant pathways include the cytosolic DNA sensing pathway (Figure 5B) and the Gɑ_12/13_ signaling pathway (Figure 5D).

### 3.6. Proposed Two-Stage Diagnostic Algorithm for miRNA-Based Classification of Small B-Cell Lymphomas

**Figure 6:**
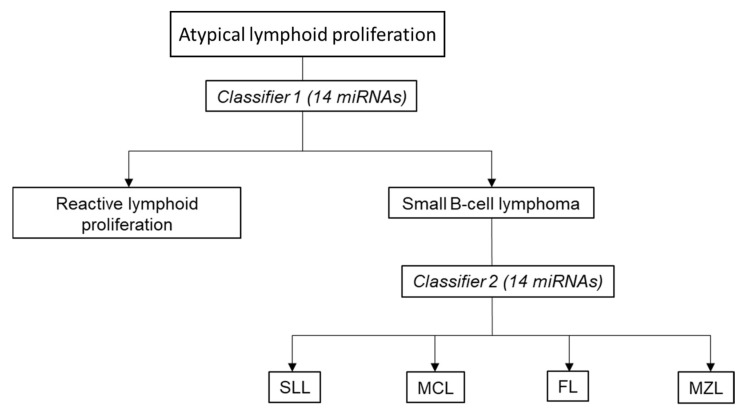
A proposed diagnostic algorithm incorporating miRNA-based classifiers for the workup of lymphoid proliferation morphologically suspicious of small B-cell lymphoma. Abbreviations: miRNA, microRNA; SLL, small lymphocytic lymphoma/chronic lymphocytic leukemia; CLL, chronic lymphocytic leukemia; FL, low-grade follicular lymphoma; MCL, mantle cell lymphoma; MZL, marginal zone lymphoma.

Given that distinguishing small B-cell lymphoma from reactive lymphoid proliferation represents a frequent diagnostic dilemma confronting many practicing pathologists, herein we propose a two-staged algorithm where miRNA-based classifiers instead of a wide panel of IHC markers is used to diagnose and subtype lymphoid proliferations that are morphologically suspicious of small B-cell lymphoma (Figure 6).

## 4. Discussion

### 4.1. miRNAs as Potential Diagnostic Biomarkers

In this era of individualized medicine, precise diagnosis and subtype classification of lymphomas have become increasingly important with the availability of disease-specific protocols and new targeted agents that inhibit specific pathways for different lymphoma subtypes. However, practicing lymphoma pathology is highly challenging. Accurate lymphoma diagnosis often requires the availability of hematopathologists with deep knowledge and experience in evaluating lymphoid lesions, high-quality laboratory infrastructure, as well as easy accessibility to a wide panel of immunohistochemical stains and additional molecular genetic testing such as fluorescence in situ hybridization (FISH) and clonality studies, all of which may not be available in resource constrained nations. In addition, pathologists have to make do with increasingly smaller samples, which do not permit the application of a large number of immunostains. Given that small B-cell proliferation is one of the most common lymphoid lesions encountered by general pathologists, we investigated the biomarker potential of miRNA expression signatures as a diagnostic adjunct to improve the identification and subtyping of small B-cell lymphoma. We used a novel high throughput qPCR platform to develop miRNA-based classifiers to distinguish neoplastic from reactive lymphoid proliferation and to subtype the four most common histological subtypes of small B-cell lymphomas. We then proposed a two-staged diagnostic process where the miRNA classifiers are incorporated to complement morphological evaluation (Figure 6). 

MiRNAs have previously been reported to be aberrantly expressed in almost all human cancers [16], including B-cell lymphomas [17,18]. Many of the miRNAs that have been identified thus far as potential biomarkers in lymphomas play key regulatory roles in normal B-cell development, and when dysregulated, drive lymphomagenesis which contributes to these hematological malignancies [19,20]. As active players in tumor pathogenetic pathways, miRNAs should have a significant influence on cancer diagnosis and prognosis. In fact, miRNA expression profiles have been reported by many investigators to be useful in tumor classification and subtyping [21,22,23,24,25,26], particularly in the setting of poorly differentiated malignancies and small biopsy samples where traditional morphological and antigenic evaluation have proven to be difficult if not impossible; while others have identified miRNA signatures associated with disease prognosis [21,27,28,29] and response to treatments [30,31,32,33]. 

The application of miRNA expression profiling in the field of molecular cancer diagnostics requires a practical and reliable method that works on routinely available clinical materials. miRNAs can be robustly detected in FFPE tissue samples because they are small and less susceptible to degradative processes, and have been reported to be stable in FFPE archival tissue specimens that have been stored for close to 30 years [34]. Remarkably, other investigators have reported the superiority of miRNAs as analytes compared with mRNAs for the molecular characterization of compromised archived clinical specimens [35] and in the accurate classification of metastatic cancers of unknown primary origins [25,36]. In addition, it has been shown that the miRNA composition in frozen tissues is largely preserved and comparable to that of routinely fixed (6–24 h) FFPE tissue specimens [37]. These studies highlight the adequacy, feasibility, and exciting potential of using miRNAs in archival FFPE tissue samples as novel clinical biomarkers.

Although miRNAs have unique attributes that render them suitable biomarkers in clinical practice, their accurate detection and quantification can be challenging because of their small size and sequence similarity among various members. For biomarker discovery and genome-wide expression analyses, most investigators deployed high-throughput hybridization-based methods, such as microarray technology for global gene expression profiling [38]. Although microarray technology is a powerful approach that enables simultaneous screening of large numbers of miRNAs, its performance is most robust when frozen tissue or freshly fixed FFPE tissue are used, as prolonged storage of FFPE tissue blocks (up to 11 years) leads to a significant drop in miRNA detection [37]. Other miRNA detection methods, including in situ hybridization and next-generation sequencing, are technically more challenging. Barriers to clinical adoption include higher costs, need for sophisticated instrumentation, and complicated data interpretation.

The current gold standard for quantitative gene expression measurement is quantitative PCR. qPCR is a robust and reproducible technology that can detect low levels of miRNAs with high sensitivity and specificity [39,40], and it is widely used by investigators to validate genome-wide miRNA expression data derived from other techniques [41], especially for the analysis of a small panel of miRNAs. The efficiency of this technique in archived FFPE specimens has also been adequately demonstrated. PCR-based miRNA profiling platforms require much lower RNA input compared with other quantification methods, which is clearly a key advantage when dealing with limited and often compromised clinical specimens. Moreover, being a well-established technology, one key advantage of qPCR is that it can be easily and conveniently performed in most clinical diagnostic laboratories (especially after the COVID pandemic), and it produces data that are easy to analyze. Therefore, validation of a PCR-based laboratory-developed test (LDT) for accreditation purposes is likely to be far less complex compared to other more sophisticated platforms. 

The main challenge of PCR-based miRNA biomarkers discovery work lies in the design of individual primers required for specific amplification of each miRNA gene included in large-scale analyses. Due to the short length of miRNAs (roughly the size of a PCR primer), primer design for specific PCR amplification poses significant difficulty. As such, most commercially available high throughput qPCR platforms employ only one or two miRNA-specific primers with selective incorporation of universal primers. In the current study, we performed multiplex comparative analyses of 360 miRNAs based on a unique method that uses three miRNA-specific primers (i.e., stem-loop RT, forward and reverse primers), obviating the use of universal primers altogether. All the primers of each miRNA analyzed have been carefully designed to detect single nucleotide differences. We believe that this platform offers a unique advantage to detect both low- and high-abundance miRNAs with unparalleled specificity. The combination of high sensitivity and specificity, broad dynamic range and multiplexing capability of this assay hold great promise in delivering highly reliable, reproducible, and representative disease-specific miRNA profiles. 

With this novel miRNA RT-qPCR profiling platform, we found two unique miRNA-based classifiers, each comprising a small set of 14 miRNAs, that can help to diagnose and subtype the four most common entities of small B-cell lymphomas with reasonably high accuracy. 

We believe that the proposed two-staged diagnostic workflow incorporating miRNA-based classifiers can potentially serve as a cost-effective and practical tool to complement traditional morphological diagnosis, especially in the resource-constrained nations. Typically, when confronted with the differential diagnoses of reactive lymphoid hyperplasia versus one of the low-grade B-cell lymphomas, pathologists will order a panel of 7–8 immunostains, sometimes with additional fluorescence in situ hybridization (FISH) and B-cell clonality analyses. Using a curated panel of miRNA targets, the cost of our RT-qPCR assay is economical, especially when it is reactive in nature and only classifier 1 is needed (Table 2). The turnaround time of within a day also compares favourably to immunohistochemistry, FISH and clonality analysis.

Conventional wisdom may hold that IHC may be easier to perform and accessible compared to molecular techniques. In fact, optimization and validation of IHC is technically challenging and the range of antibodies available is limited due to cost constraints in developing nations. On the other hand, due to the need for COVID testing during the global COVID-19 pandemic, RT-qPCR machines have become widely available even in countries with limited resources, hence rendering our assay feasible in such countries. 

Our study has its limitations. The number of MZL and FL cases are more than three times greater than that of SLL and MCL cases for the validation cohort, even though the number of cases for each subtype in the discovery cohort is roughly the same (Table 1). Our miRNA subtype classifier has a sensitivity of 85.2–87.8% for all four subtypes despite this skewed subtype ratio in the validation cohort. However, it remains to be seen if increasing the number of samples for SLL and MCL will further augment the sensitivity of the resultant miRNA classifier. Additionally, our miRNA classifiers are developed based on the patient cohort of a particular demographic region and it is unclear if they will achieve comparable diagnostic sensitivity for cases from other regions. 

### 4.2. Biological Relevance of miRNA Biomarkers

Besides their potential utility as biomarkers, deregulated miRNAs in the tumors may point us to relevant cancer biology under their regulation and provide insights into B-cell lymphomagenesis and progression. Most of the top 14 differentially expressed miRNAs between the small B-cell lymphomas and reactive lymphoid samples in our study are in concordance with existing literature.

The downregulation of miR-29b-2-5p in our samples corroborates a finding on the repression of miR-29, a tumor suppressor, in mantle cell lymphoma and other aggressive Myc-driven lymphomas [42]. This repression is mediated by MYC, through the epigenetic modifiers histone deacetylase 3 (HDAC3) and histone-lysine N-methyltransferase 2 (EZH2). Additionally, overexpression or translocation of MYC is a common theme across many types of lymphomas, including more aggressive variants of FL, MCL, and SLL/CLL [43]. Another highly relevant miRNA is miR-9, reported to be upregulated in FL patients [44], and is also upregulated in our B-cell lymphoma samples. The pathogenic role of miR-9 lies in its downregulation of PRDM1, an important transcription factor in the terminal differentiation of B cells [44]. Another miRNA that is downregulated in MCL samples and also reduced in our small B-cell lymphoma samples is miR-223-3p [45]. This miRNA reduces proliferation and enhances apoptosis in MCL cells by targeting the CHUK/NF-κB2 signaling axis [45].

Several differentially expressed miRNAs are in concordance with studies done on other hematological malignancies that may share common underlying mechanisms in lymphomagenesis. For instance, miR-342-5p reduces cancer cell proliferation by targeting a cell cycle regulator Cyclin D1 (encoded by the CCND1 gene), and is reported to be downregulated in chronic myeloid leukemia patients [46]. Similarly, miR-342-5p is reduced in our lymphoma samples. CCND1 is highly relevant in our study as the t(11;14) translocation of CCND1 to the immunoglobulin heavy chain is a hallmark of early MCL transformation [47]. Our findings appear to suggest that the downregulation of miR-342-5p may also contribute to the overexpression of Cyclin D1 in lymphomagenesis.

MiR-139-5p has been reported to be a tumor suppressor by repressing EIF4G, a factor in the initiation of protein translation, and is downregulated across various subtypes of acute myeloid leukemia (AML) [48]. EIF4G, the target of miR-139-5p repression, is also highly activated in lymphoma cells, specifically diffuse large B-cell lymphoma (DLBCL), pointing to the possibility of miR-139-5p suppression across hematological malignancies [49]. In line with these studies, miR-139-5p is also downregulated in our lymphoma samples. 

Yet, another tumor suppressor, miR-126, suppresses T and B-cell migration—a key lymphomagenesis event—by targeting the G protein-coupled receptor (GPCR), S1PR2 [50]. Low expression of miR-126 is also correlated with worse overall survival in angioimmunoblastic T-cell lymphoma (AITL), hence asserting its relevance in lymphomagenesis and progression. Another related GPCR, S1PR1, is also targeted by miR-145-5p, a tumor suppressor reported in DLBCL [51]. In line with these findings, the tumor-suppressing miR-126-5p and miR-145-5p are also downregulated in our lymphoma samples.

Additionally, in DLBCL, a circular RNA circ_0003645 acts as a miRNA sponge to miR-335-5p, hence abolishing miR-335-5p’s tumor suppressive effect in targeting NFIB [52]. The downregulation of miR-335-5p in our samples may achieve a similar effect in enhancing the expression of NFIB. Importantly, NFIB is identified as a hub gene that is overexpressed across many lymphoma RNA-sequencing datasets [53].

To the best of our knowledge, not much is known yet about miR-143-5p and miR-224-5p—both downregulated in our lymphoma samples. However, studies by association appear to correlate miR-143 polymorphisms to reduced incidence of non-Hodgkin lymphomas in caucasian populations [54] and high miR-224-5p expression to better survival outcomes in DLBCL patients, indicating possible underlying mechanisms [55].

### 4.3. Possible Pathways Implicated by miRNAs

#### 4.3.1. Cytosolic DNA Sensing Pathway, RIG-I-like Receptor Signaling Pathway and NOD-like Receptor Signaling Pathway

With the ability to bind to thousands of target mRNAs by complementary base pairing, miRNAs could potentially play diverse regulatory roles in many processes, including tissue and cancer development like lymphomagenesis. 

Interestingly, three highly significant and upregulated KEGG pathways—cytosolic DNA sensing pathway, RIG-I-like receptor signaling pathway, and NOD-like receptor signaling pathway—are functionally related. These pathways underlie the sensing of foreign matter that may be introduced during infections, in the form of single or double-stranded DNA, from viruses and other pathogens [56]. Hence, these pathways are particularly relevant in lymphomagenesis where infection by oncogenic viruses, such as EBV and KSHV, and other pathogens like bacteria can transform B cells into lymphomas in certain cases [57]. Gastric MALT, a type of MZL, has been observed to arise along with chronic gastritis caused by Helicobacter pylori [58] and regress upon antibiotics treatment, suggesting an infection-driven tumorigenic event [59]. Another example, another type of MZL, ocular adrenal MALT, has been linked to Chlamydophila psittaci infection [60]. 

While molecular mechanisms linking infection and these pathways are still unclear in the context of small B-cell lymphoma, these pathways have been known to eventually lead downstream to NF-κB signaling, which is heavily implicated in lymphomagenesis [61]. Constitutive NF-κB activation is a hallmark of B-cell lymphomagenesis, thus genetic alterations and pathways that drive its activation are of high clinical and therapeutic value. Hence, these pathways may constitute novel, clinically relevant upstream players in B-cell lymphomagenesis. 

Additionally, inactivating somatic mutations or deletions have been reported in the tumor suppressor gene, ubiquitin-editing protein A20 (or TNFAIP3) across many types of lymphomas including MALT and FL [62]. Overexpression of A20 has been shown to attenuate RIG-I signaling [63]. Restoration of wild-type A20 in A20-inactivated lymphoma cell lines also led to the repression of NF-κB signaling [64], suggesting that A20 is a regulator of the RIG-I and NF-κB signaling axis. Hence, these findings appear to further support a potential link between the upregulation of the RIG-I-like signaling pathway to small B cell lymphomas. 

Interestingly, a recent study reported on the absence of the expression of STING (a part of the cGAS-STING, which are major components of the cytosolic DNA sensing pathway) specifically in B-cell non-Hodgkin lymphomas, including various small B-cell lymphomas like FL, MCL, MZL and SLL, but not in T- and NK-cell lymphomas [65]. This finding may point to the involvement of other components in the pathway that may have not yet been studied in small B-cell lymphomas. 

#### 4.3.2. Gɑ_12/13_ Signaling Events

The G12 subfamily, of which Gɑ_12_ and Gɑ_13_ are members, consist of G proteins, which are G-alpha subunits of heterotrimeric GTP-binding proteins. G proteins serve as the intermediary between GPCRs on the cell membrane and downstream signaling, and they work by binding to guanine nucleotides. G12 proteins, together with other G protein subfamilies, form the most diverse group of receptors, playing a wide range of important roles in normal physiology. In the context of this study, Gɑ_12_ and Gɑ_13_ have been demonstrated to regulate the maturation of B cells in the marginal zone in a murine model [66]. Unsurprisingly, the overexpression or enhanced activation of Gɑ_12_ and Gɑ_13_ has been linked to several cancers. However, G12 proteins still remain one of the least understudied subfamily in cancer biology, especially in hematological malignancies. However, the few studies done in lymphomas do point towards significant roles that Gɑ_12/13_ signaling play in lymphomas in general [66,67,68]. 

Constitutive NF-κB signaling is known as an important hallmark of lymphomas and much work has been done on pathways that drive its activity. Enhanced NF-κB activity has been associated with increased hedgehog signaling. Smoothened (SMO), yet another GPCR and also an essential signal transducer of the hedgehog signaling pathway, has been shown to recruit and activate Gɑ_i_ and Gɑ_12_, and not other G proteins. The resulting signaling complex then initiates a cascade of events involving non-canonical signaling complexes, ultimately leading to the activation of NF-κB signaling [67]. This study suggests that Gɑ_12_ could play an important enabling role in lymphomagenesis by mediating the activation of NF-κB signaling.

Conversely, Gɑ_13_, along with associated receptors S1PR2 and P2RY8, appear to promote the confinement of B cells to ensure a physiologically normal germinal center. Gɑ_13_ deficiency has been shown to give rise to germinal center B-cell-derived lymphoma in mice [68]. Similarly, mutations in GNA13 (the gene encoding Gɑ_13_), S1PR2, or P2RY8—found in GCB-DLBCL patients—have been demonstrated to cause the dissemination of germinal center B-cells (and in the case of P2RY8 mutations, also enhancing cell growth), hence also leading to germinal center B-cell-derived lymphoma [68]. Unlike Gɑ_12_, Gɑ_13_ plays a tumor suppressive role in orchestrating the proper development of the germinal center.

Taking together the limited knowledge gathered on Gɑ_12/13_ signaling events in lymphomas, we hypothesize that Gɑ_12_ signaling could be a significant player in small B-cell lymphomagenesis.

#### 4.3.3. Other Notable Signaling Pathways

miRSEA analysis also identified pathways that are regularly implicated in lymphomas, hence validating the relevance of biomarker miRNAs that differentiate small B-cell lymphomas from RL [69]. Significantly upregulated pathways (with q-value of less than 0.05) include the B-cell receptor signaling pathway, mTOR signaling pathway, and PI3K-Akt activation (Supplementary File S1).

## 5. Conclusions

Overall, our results demonstrate that miRNA expression profiling may serve as a promising biomarker and practical tool to aid the diagnosis of common lymphoid lesions. Specifically, we identified and validated two unique miRNA-based classifiers that can help to diagnose and subtype the four most common diagnostic entities of small B-cell lymphomas. 

## Figures and Tables

**Table 1:** Tissue sites of recruited samples in discovery and validation cohorts.

		Tissue Sites	
	Subtypes	Nodal (*n*)	Extranodal (*n*)
Discovery Cohort	SLL	20	3
	FL	5	16
	MCL	12	8
	MZL	0	19
	RL	15	2
Validation Cohort	SLL	14	6
	FL	53	21
	MCL	13	9
	MZL	5	69
	RL	54	38

Extranodal sites include oropharyngeal mucosa, respiratory tract, gastrointestinal tract, bladder, salivary gland, eye, thyroid, and skin tissues. SLL = small lymphocytic lymphoma/chronic lymphocytic leukemia; MCL = mantle cell lymphoma; FL = low-grade follicular lymphoma; MZL = marginal zone lymphoma. RL = reactive lymphoid.

**Table 2:** Comparison between our proposed RT-qPCR assay and traditional IHC/FISH for small B-cell lymphoma classification.

	Proposed RT-qPCR Assay	IHC and FISH
Cost (based on charges in our institution)	USD115-150 (classifier 1) USD230-300 (classifier 1 + 2)	IHC with 7 antibodies: USD605 BCL2 FISH: USD375 B-cell clonality: USD680
Turnaround time	Within a day	IHC: 1–2 days FISH: 5–7 days Clonality analysis: 7–10 days

## Data Availability

The datasets used and/or analysed during the current study are available from the corresponding author on reasonable request.

## Supplementary Materials

The following supporting information can be downloaded at: https://www.mdpi.com/article/10.3390/cancers15020453/s1, Supplementary File S1: miRSEA analysis.
